# Design, Synthesis, and Spectral Properties of Novel 2-Mercaptobenzothiazole Derivatives

**DOI:** 10.3390/ma17010246

**Published:** 2024-01-02

**Authors:** Agnieszka Skotnicka, Janina Kabatc-Borcz

**Affiliations:** Faculty of Chemical Technology and Engineering, Bydgoszcz University of Science and Technology, Seminaryjna 3, 85-326 Bydgoszcz, Poland; nina@pbs.edu.pl

**Keywords:** 2-mercaptobenzothiazole, synthesis, spectroscopic properties, quantum chemical calculations

## Abstract

This paper is focused on the optimalization of methods for the synthesis, isolation, and purification of 2-mercaptobenzothiazole-based acrylic and methacrylic monomers. The structures of the newly synthesized compounds were confirmed through infrared (IR) and nuclear magnetic resonance spectroscopy (NMR). Spectroscopic properties of the resulting 2-mercaptobenzothiazole derivatives were determined based on their absorption spectra and molar absorption coefficients in solvents with varying polarities. A correlation was established between the calculated density functional theory (DFT) energies and Frontier Molecular Orbitals and the experimental observations, confirming their consistency. The practical utility of the synthesized compounds, particularly in future polymerization processes, hinges on a thorough understanding of these properties.

## 1. Introduction

**Figure 1:**
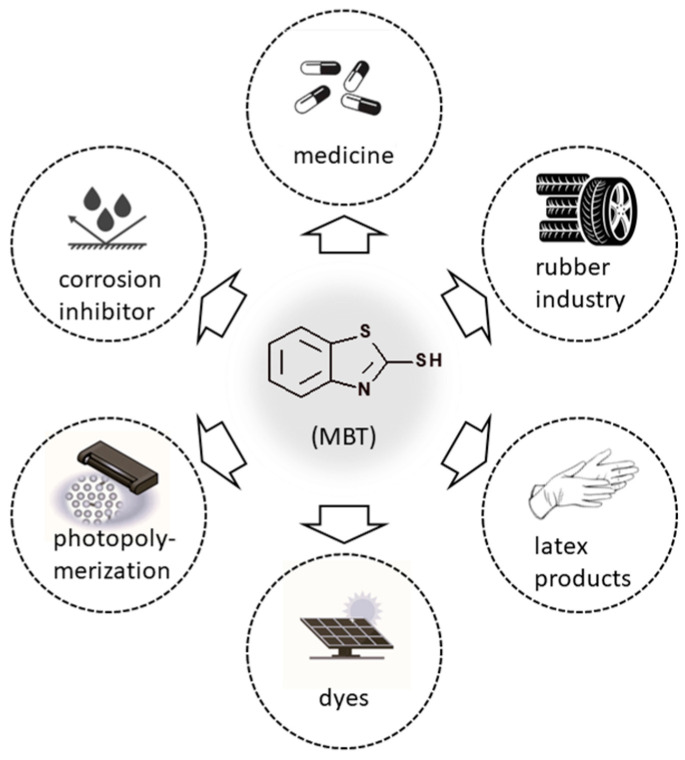
Examples of applications of 2-mercaptobenzothiazole.

The 2-mercaptobenzothiazoles (MBT) (Figure 1) constitute a significant group of bioactive and industrially crucial organic compounds. It is noteworthy that their derivatives are of huge interest in scientific research owing to their potential pharmacological properties [1]. The widespread use of these substances in the medical field, such as their application as anti-inflammatory [2], antibacterial [3,4], and antifungal agents [5], along with their efficacy in treating diseases such as cancer and cardiovascular illnesses [6], has garnered considerable attention. The interest in this group of compounds arises not only from their medicinal applications but also from their diverse uses in various industries: 2-mercaptobenzothiazole serves as a widely utilized organic corrosion inhibitor [7] for copper [8,9] and zinc [10,11], and as a component of self-healing and anticorrosion smart coatings [12,13,14,15]. Larsson et al. [16] suggested that MBT can serve as an additive in dye solar devices. Moreover, 2-mercaptobenzothiazole and its derivative zinc 2-mercaptobenzothiazole (ZMBT) [17] are commonly used as ‘hemi-ultra’ accelerators in the vulcanization process of both natural and synthetic rubber. Additionally, they find applications as plasticizers in the rubber industry [18,19,20]. Heteroaromatic thiols, including MBT, among others, have been utilized as co-initiators for type II photo-initiators composed of camphoroquinone and isopropylthioxanthone. Kinetic data indicate their efficiency as co-initiators, comparable to aromatic amines. Due to their ability to readily donate hydrogen atoms, some of these thiols have been employed as co-initiators of hexaarylbisimidazoles (HABI) [21]. In other study, Degirmenci et al. copolymerized a methacrylic monomer containing a benzothiazole disulfide group with a hydrophilic polyethylene glycol (PEG)-based monomer, resulting in a redox-responsive functional polymer. The straightforward synthesis of these copolymers, which are highly functionalizable, and their application in the preparation of reversibly functionalizable coatings, establishes a versatile interface for a range of biomedical applications [22].

Acrylic acid derivatives, commonly referred to as acrylates, encompass salts and esters derived from acrylic and methacrylic acid. Characterized by a vinyl group linked to a carboxyl group, these compounds undergo facile polymerization owing to an active double bond within their structure. As monomers, they rank among the most widely employed in the synthesis of polymeric plastics. The inherent compatibility of acrylic and methacrylic monomers facilitates polymerization not only with each other but also with diverse monomers, thereby contributing to the fabrication of numerous polymer compositions [23,24]. Manipulating the composition and proportions allows for the production of materials with varied final properties [25]. Acrylic polymers, renowned for their diverse properties, encompassing excellent durability, transparency, strength, UV stability, ease of dyeing, and color fastness, find extensive utility in the production of various commodities. These polymers play pivotal roles in optics [26] and medicine [27,28] and numerous industries, notably in the manufacturing of paints and adhesives [29], for the design of macromolecular self-organizing materials [30,31], as well as applications in the textile industry. Their versatility extends across a spectrum of forms, ranging from very soft adhesive materials to loose powders, and even to rigid, hard boards and consumer products.

**Figure 2:**
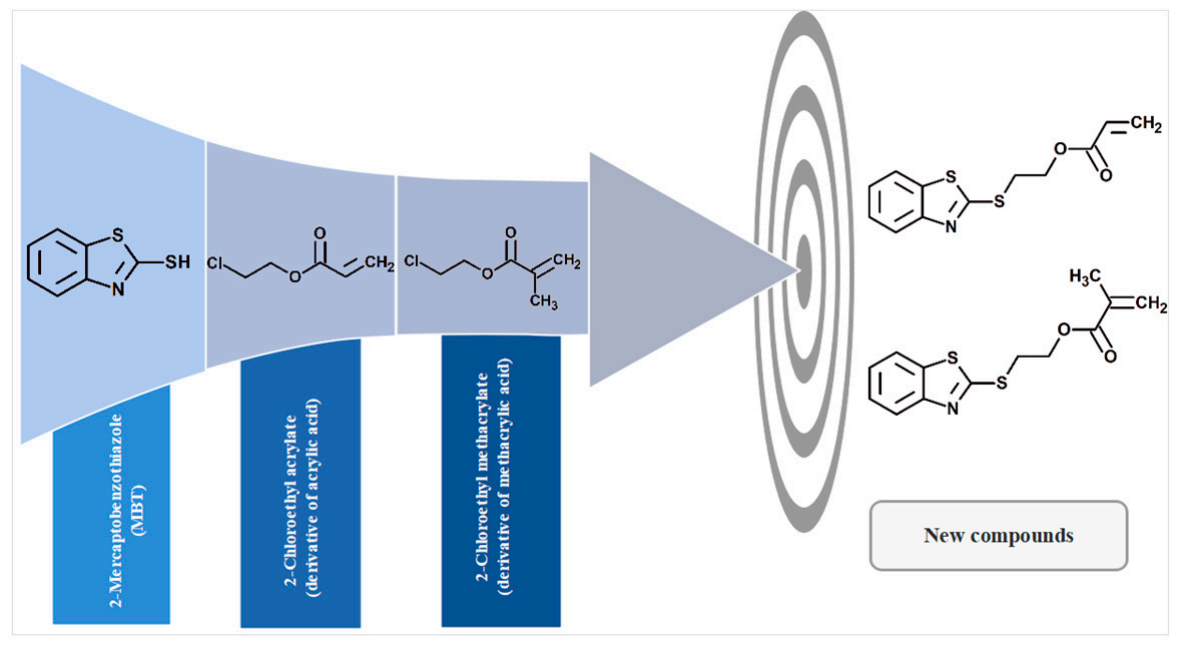
New compounds based on 2-mercaptobenzothiazole and derivatives of (meth)acrylic acid.

The distinctive characteristics of 2-mercaptobenzothiazole and its acrylic and methacrylic acid derivatives motivated the development of a novel group of compounds (Figure 2). These newly synthesized compounds can be regarded as functional monomers, showing potential applications in the expansive domains of polymer chemistry and technology. The objectives of this paper is the synthesis and study on the structures and spectroscopic properties of the new compounds based on 2-mercaptobenzothiazole. It is assumed that the indicated acrylate and methacrylate derivatives of 2-mercaptobenzothiazole have the potential to serve as photo-initiators of radical polymerization of various acrylate monomers. Furthermore, some of these compounds can be used as photo-initiators, as well as serve as attractive building units and components for advanced polymeric materials with a high refractive index. Examples of such applications include their use in foldable intraocular lenses [32] and for industrial applications, such as coatings [33,34].

## 2. Materials and Methods

### 2.1. Materials

All the reagents and solvents were purchased from Sigma-Aldrich (Poznań, Poland) and utilized without additional purification. The spectroscopic studies necessitated the use of chemicals with the highest purity (≥99%).

### 2.2. Synthesis

The synthesis followed the methodology outlined in [33]. The method of purification was modified. Substituted 2-mercaptobenzothiazole (28 mmol) and sodium bicarbonate (28.5 mmol, 2.4 g) were dissolved in 10 mL of dimethylformamide at 60 °C. Then, 2-chloroethyl acrylate (29 mmol, 3.47 mL) or 2-chloroethyl methacrylate (29 mmol, 2.93 mL) was added, dropwise. The reaction mixture was refluxed overnight, washed with a 5% NaOH aqueous solution, and extracted with diethyl ether. The organic layer was separated and dried using MgSO_4_, filtered, and the solvent was removed under vacuum. The crude oil underwent purification through silica gel column chromatography using dichloromethane as the eluent, with fractions collected at 285 nm.

#### Elemental Analysis

Elemental analysis was as follows:

2-(2-(6-Methylbenzothiazolyl)thio)ethyl acrylate (**1**). Bp 238.7 °C, yellow oil. IR (ATR), cm^−1^: 3031–3425, 2860–2951, 1720, and 1632. λ_abs_: 283 nm (DCM). ^1^H NMR (400 MHz, CDCl_3_) δ (ppm): 7.72 (d, 1H, ^3^*J*_H,H_ = 8.28 Hz, Ar), 7.51 (s, 1H, Ar), 7.21 (m, 1H, Ar), 6.41 (d, 1H, ^3^*J*_H,H_ = 17.32 Hz, CH=CH_2_), 6.11 (q, 1H, CH=CH_2_), 5.82 (d, 1H, ^3^*J*_H,H_ = 10.48 Hz, CH=CH_2_), 4.53 (t, 2H, CH_2_–O), 3.63 (t, 2H, S–CH_2_), 2.39 (t, 3H, CH_3_). ^13^C NMR (100 MHz, CDCl_3_) δ (ppm): 165.8, 164.1, 151.1, 135.5, 134.6, 131.2, 128.0, 127.6, 121.1, 120.9, 62.3, 31.9, 21.4. C_13_H_13_NO_2_S_2_, Calcd. C, 55.89, H, 4.69, N, 5.01, S, 22.95. Found C, 55.45, H, 6.70, N, 4.83, S, 23.36, M^+^: 279.1 (Supplementary Materials).

2-(2-(6-Chlorobenzothiazolyl)thio)ethyl acrylate (**2**). Bp 251.9 °C, light orange oil. IR (ATR), cm^−1^: 3036–3435, 2690–2946, 1720, and 1633. λ_abs_: 285 nm (DCM). ^1^H NMR (400 MHz, CDCl_3_) δ (ppm): 7.67 (d, 1H, ^3^*J*_H,H_ = 8.68 Hz, Ar), 7.63 (d, 1H, ^3^*J*_H,H_ = 1.92 Hz, Ar), 7.29 (dd, 1H, Ar), 6.34 (d, 1H, ^3^*J*_H,H_ = 17.32 Hz, CH=CH_2_), 6.04 (q, 1H, CH=CH_2_), 5.76 (d, 1H, ^3^*J*_H,H_ = 10.44 Hz, CH=CH_2_), 4.46 (t, 2H, CH_2_–O), 3.58 (t, 2H, S–CH_2_). ^13^C NMR (100 MHz, CDCl_3_) δ (ppm): 166.2, 165.7, 151.6, 136.5, 131.3, 130.2, 128.0, 127.8, 122.2, 120.6, 62.6, 31.9. C_12_H_10_ClNO_2_S_2_, Calcd. C, 48.08, H, 3.36, N, 4.67, S, 21.39. Found C, 48.2, H, 3.56, N, 4.59, S, 21.15, M^+^: 299.0 (Supplementary Materials).

2-(2-(5-Chlorobenzothiazolyl)thio)ethyl acrylate (**3**). Bp 252.4 °C, yellow oil. IR (ATR), cm^−1^: 3035–3435, 2658–2947, 1721, and 1634. λ_abs_: 279 nm (DCM). ^1^H NMR (400 MHz, CDCl_3_) δ (ppm): 7.76 (s, 1H, Ar), 7.56 (d, 1H, ^3^*J*_H,H_ = 8.48 Hz, Ar), 7.19 (d, 1H, ^3^*J*_H,H_ = 7.8 Hz, Ar), 6.35 (d, 1H, ^3^*J*_H,H_ = 17.32 Hz, CH=CH_2_), 6.04 (q, 1H, CH=CH_2_), 5.77 (d, 1H, ^3^*J*_H,H_ = 10.44 Hz, CH=CH_2_), 4.47 (t, 2H, CH_2_–O), 3.60 (t, 2H, S–CH_2_). ^13^C NMR (100 MHz, CDCl_3_) δ (ppm): 167.7, 165.6, 153.7, 133.5, 132.0, 131.2, 127.9, 124.6, 121.5, 121.3, 62.4, 31.7. C_12_H_10_ClNO_2_S_2_, Calcd. C, 48.08, H, 3.36, N, 4.67, S, 21.39. Found C, 48.25, H, 3.53, N, 4.0, S, 21.66, M^+^: 299.0 (Supplementary Materials).

2-(2-(6-Methylbenzothiazolyl)thio)ethyl methacrylate (**4**). Bp 248.1 °C, dark brown oil. IR (ATR), cm^−1^: 2950–3021, 2734–2922, 1715, and 1636. λ_abs_: 285 nm (DCM). ^1^H NMR (400 MHz, CDCl_3_) δ (ppm): 7.65 (d, 1H, ^3^*J*_H,H_ = 8.08 Hz, Ar), 7.44 (s, 1H, Ar), 7.12 (dd, 1H, Ar), 6.03 (s, 1H, C(CH_3_)=CH_2_), 5.47 (s, 1H, C(CH_3_)=CH_2_), 4.44 (t, 2H, CH_2_–O), 3.56 (t, 2H, S–CH_2_), 2.36 (s, 3H, CH_3_), 1.83 (s, 3H, C(CH_3_)=CH_2_). ^13^C NMR (100 MHz, CDCl_3_) δ (ppm): 167.0, 164.2, 151.2, 136.0, 135.5, 134.5, 127.5, 126.0, 121.1, 120.8, 62.9, 31.9, 21.4, 18.2. C_14_H_15_NO_2_S_2_, Calcd. C, 57.31, H, 5.15, N, 4.77, S, 21.86. Found C, 57.01, H, 5.30, N, 5.07, S, 21.71, M^+^: 293.1 (Supplementary Materials).

2-(2-(6-Chlorobenzothiazolyl)thio)ethyl methacrylate (**5**). Bp 274.1 °C, brown oil. IR (ATR), cm^−1^: 2951–3021, 2734–2922, 1715, and 1636. λ_abs_: 285 nm (DCM). ^1^H NMR (400 MHz, CDCl_3_) δ (ppm): 7.78 (d, 1H, ^3^*J*_H,H_ = 8.68 Hz, Ar), 7.75 (d, 1H, ^3^*J*_H,H_ = 2.08 Hz, Ar), 7.40 (dd, 1H, Ar), 6.13 (s, 1H, C(CH_3_)=CH_2_), 5.58 (s, 1H, C(CH_3_)=CH_2_), 4.55 (t, 2H, CH_2_–O), 3.69 (t, 2H, S–CH_2_), 1.94 (s, 3H, C(CH_3_)=CH_2_). ^13^C NMR (100 MHz, CDCl_3_) δ (ppm): 167.0, 166.4, 151.6, 136.5, 135.9, 130.3, 126.8, 126.0, 122.2, 120.6, 62.7, 32.0, 18.2. C_13_H_12_ClNO_2_S_2_, Calcd. C, 49.75, H, 3.85, N, 4.46, S, 20.44. Found C, 49,90, H, 4.00, N, 4.31, S, 20.29., M^+^: 313.0 (Supplementary Materials).

2-(2-(5-Chlorobenzothiazolyl)thio)ethyl methacrylate (**6**). Bp 280.7 °C, orange oil. IR (ATR), cm^−1^: 3066–3085, 2836–2978, 1714, and 1636. λ_abs_: 279 nm (DCM). ^1^H NMR (400 MHz, CDCl_3_) δ (ppm): 7.77 (s, 1H, Ar), 7.57 (d, 1H, ^3^*J*_H,H_ = 8.48 Hz, Ar), 7.20 (dd, 1H, Ar), 6.04 (s, 1H, C(CH_3_)=CH_2_), 5.49 (s, 1H, C(CH_3_)=CH_2_), 4.46 (t, 2H, CH_2_–O), 3.60 (t, 2H, S–CH_2_), 1.85 (s, 3H, C(CH_3_)=CH_2_). ^13^C NMR (100 MHz, CDCl_3_) δ (ppm): 167.9, 167.0, 153.8, 135.9, 133.5, 132.2, 126.0, 124.7, 121.6, 121.4, 62.7, 31.9, 18.2. C_13_H_12_ClNO_2_S_2_, Calcd. C, 49.75, H, 3.85, N, 4.46, S, 20.44. Found C, 49,40, H, 4.14, N, 4.17, S, 20.79, M^+^: 313.0 (Supplementary Materials).

### 2.3. Experimental Measurements

#### 2.3.1. NMR Measurements

The ^1^H and ^13^C NMR spectra were measured on a Bruker Ascend III spectrometer operating at 400 MHz (Bydgoszcz, Poland). Chloroform served as the solvent, and tetramethylsilane (TMS) was used as the internal standard. Chemical shifts (*δ*) are reported in ppm relative to TMS and coupling constants (*J*) are expressed in Hz.

#### 2.3.2. Elemental Analysis Measurements

Elemental analysis was performed using a Vario MACRO 11.45–0000 Elemental Analyzer System, GmbH (Toruń, Poland), with VARIOEL software (version 5.14.4.22).

#### 2.3.3. UV-Vis Measurements

The absorption spectra were measured at room temperature in a quartz cuvette (1 cm) using an Agilent Technology UV-Vis Cary 60 Spectrophotometer (Bydgoszcz, Poland).

#### 2.3.4. FT-IR Measurements

Infrared spectra were measured using an Alpha Bruker’s compact FT-IR spectrometer equipped with diamond ATR, covering the range of 4000–360 cm^−1^ (Bydgoszcz, Poland).

#### 2.3.5. Boiling Point

The boiling point was measured on the Melting Point M-565 Apparatus (Buchi; Bydgoszcz, Poland) with the following parameters: temperature gradient 5.0 °C/min, barometric pressure 1013 mbar, and boiling frequency 0.6 Hz.

#### 2.3.6. GC-MS Measurements

Chromatographic analyses were performed using an Agilent 7890B (Bydgoszcz, Poland), equipped with a split/splitless injector and multipurpose autosampler, and an Agilent 5977B mass-selective detector. The GC was fitted with a ZB-5-MS column (Zebron, Phenomenex Inc., Torrance, CA, USA), 30 m × 0.25 mm 0.25 µm, containing (5% phenyl)-methylpolysiloxane.

The injector port was held at 270 °C and used in the split mode (5:1), and 1 µL injections were made. The temperature program used for the analysis was as follows: 80 °C (2 min), ramped at 15 °C/min to 250 °C, and held for 15 min. Helium was used as the carrier gas at a flow rate of 1 mL min^−1^.

Full-scan mass spectra were recorded with an *m*/*z* range of 50–400 in electron-impact mode at 70 eV.

The transfer line and ion source temperatures were set at 280 and 230 °C, respectively. The scan rate was 2.9 scan/s, with a cathode delay time of 5 min. The SCAN mode was used for identification of analytes.

#### 2.3.7. Quantum Mechanical Calculations

Geometry optimization, vibrational spectra, and the energies of the Highest Occupied Molecular Orbital (HOMO) and Lowest Unoccupied Molecular Orbital (LUMO) were calculated using density functional theory (DFT) with the B3LYP [35,36,37] functional and 6–311+G(d,p) basis set [38,39]. The calculations were performed using Gaussian 09 software [40]. Analysis of frontier orbitals and FT-IR spectra was conducted using the Avogardo 1.2.0 application [41].

## 3. Results and Discussion

### 3.1. Synthesis and NMR Data

**Scheme 1:**
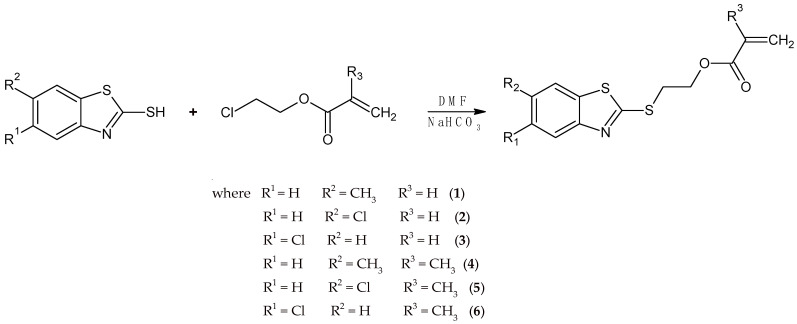
Synthesis procedure for 2-(2-benzothiazolylthio)ethyl acrylate (**1**–**3**) and methacrylate derivatives (**4**–**6**).

The 2-(2-benzothiazolylthio)ethyl acrylate derivatives (**1**–**3**) and methacrylate derivatives (**4**–**6**) presented in this paper were synthesized following the method proposed by Macaskie et al. [33], as depicted in Scheme 1.

The final products were separated and obtained in the form of oils, and were then purified via flash chromatography using dichloromethane as the eluent.

**Figure 3:**
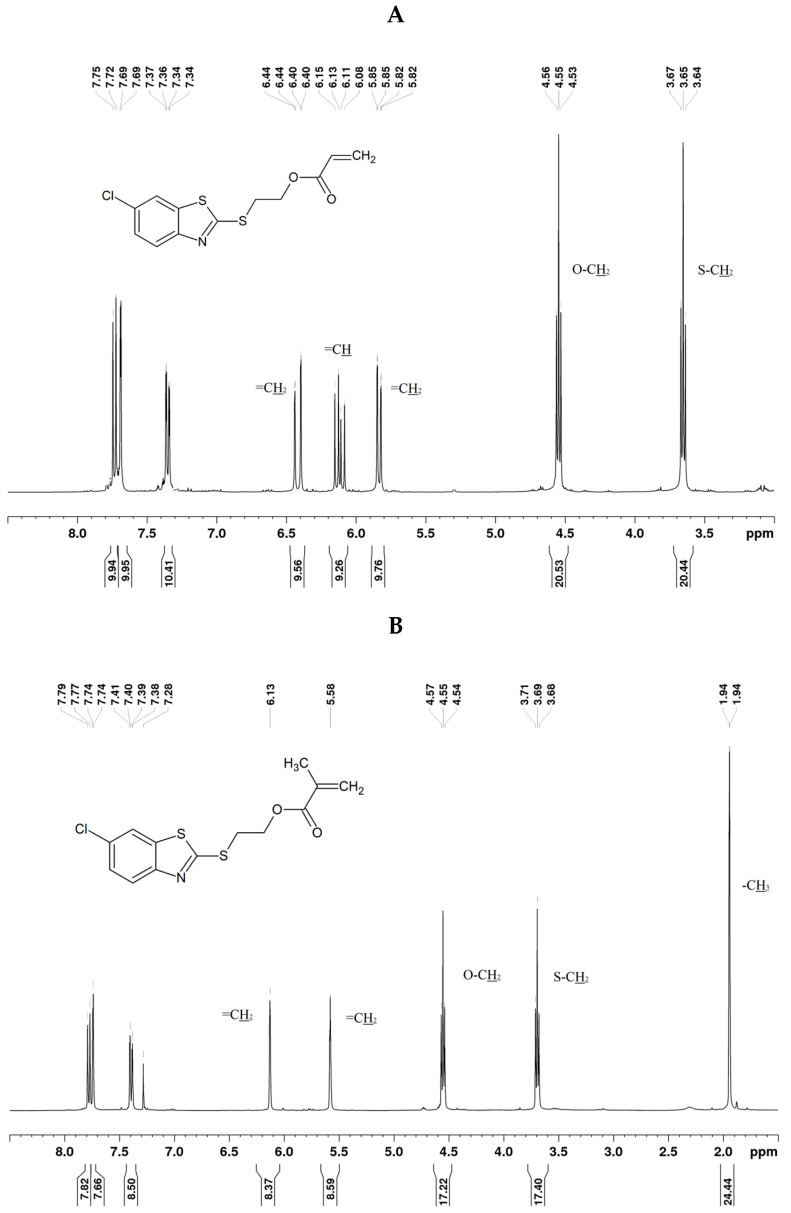
^1^H NMR spectrum (400 MHz) of 2-(2-(6-chlorobenzothiazolyl)thio)ethyl acrylate (**2**) (**A**) and 2-(2-(6-chlorobenzothiazolyl)thio)ethyl methacrylate (**5**) in CDCl_3_ (**B**).

The structures and purity of the obtained compounds were confirmed through nuclear magnetic resonance spectra, including ^1^H and ^13^C isotopes, as well as ^1^H-^13^C HMQC and ^1^H-^13^C HMBC spectra. The most characteristic chemical shifts for ^1^H and ^13^C NMR are collected in Table 1 and Table 2, respectively. The ^1^H-NMR signals for =CH_2_ in acrylic 2-mercaptobenzothiazole derivatives (**1**–**3**) appeared as doublets at δ = 5.75–6.41 ppm in CDCl_3_. The same protons in methacrylate derivatives (**4**–**6**) exhibited signals at δ = 5.47–6.13 ppm in CDCl_3_, appearing as a singlet (Figure 3). An adjacent hydrogen atom bonded to a carbon involved in the formation of a carbon–carbon double bond appeared at approximately 6 ppm as a quartet. Alkyl protons from CH_2_ groups were observed at around 4.5 ppm and 3.5 ppm for CH_2_-O and CH_2_-S, respectively. They were present as triplets in both cases. Hydrogen atoms bonded to the aromatic ring were observed in the characteristic range for aromatic protons from 7 to 8 ppm. These chemical shift values and signal patterns align with those previously reported by us for related compounds [42].

In the ^13^C NMR spectra, the most downfield peak corresponds to the carbonyl carbon atom C=O, resonating at approximately 165 ppm for acrylic (**1**–**3**) and approximately 167 ppm for methacrylic 2-mercaptobenzothiazole derivatives (**4**–**6**). On the most shielded part of the spectrum, the chemical shifts of carbons in alkane groups were observed. The chemical shifts of CH_2_-O and CH_2_-S resonated at around 63 ppm and 32 ppm, respectively (Supplementary Materials). Consistent with the ^1^H NMR spectra, these chemical shift values for all carbon atoms align with those previously described for related compounds [42].

Chemical shift values were computed to validate the experimental data. Both ^1^H and ^13^C NMR chemical shift values fell within the calculated range. The deviations observed were attributed to the common use of dimethyl sulfoxide instead of chloroform during calculations, demonstrating the impact of changing the solvent on chemical shift values.

### 3.2. Vibrational Analysis

In the identification of new chemical compounds, infrared (IR) spectroscopy serves as one of several tools. When combined with techniques such as nuclear magnetic resonance (NMR) spectroscopy, IR spectroscopy enhances the comprehensive understanding of the properties of a compound. For the study of the title compounds, vibrational spectroscopy and quantum chemical calculations using the B3LYP functional and 6–311+G(d,p) basis set were employed. Sharp bands of strong intensity, observed at 1714–1721 cm^−1^ (calculated values: 1769–1795 cm^−1^), in the analyzed of spectra of compounds were attributed to stretching vibrations of the C=O group. Additionally, in the range of 1632–1636 cm^−1^ (calculated values: 1653–1689 cm^−1^), the characteristic band of (C=C) vibrations of alkene fragments was observed. The most characteristic vibrational bands are summarized in Table 3.

### 3.3. Spectroscopic Studies

**Figure 4:**
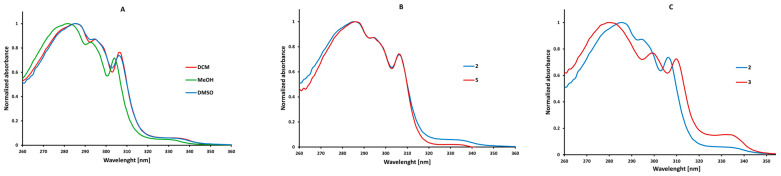
Normalized absorption spectra of: 2-(2-(6-chlorobenzothiazolyl)thio)ethyl acrylate (**2**) in solvents of different polarities (**A**), 2-(2-(6-chlorobenzothiazolyl)thio)ethyl acrylate (**2**) and 2-(2-(6-chlorobenzothiazolyl)thio)ethyl methacrylate (**5**) in dimethyl sulfoxide (**B**), and 2-(2-(6-chlorobenzothiazolyl)thio)ethyl acrylate (**2**) and 2-(2-(5-chlorobenzothiazolyl)thio)ethyl acrylate (**3**) in dimethyl sulfoxide (**C**).

The compounds under study, akin to those previously examined [42], exhibited distinct absorption in the wavelength range from 260 to 350 nm. Solvent polarity showed a weak impact on the position of maximum absorption. However, a slight shift toward a lower wavelength was noticeable when methanol was used (Figure 4A). The transition from acrylic (compounds **1**–**3**) to methacrylic (compounds **4**–**6**) did not significantly affect the position of the absorption band (Figure 4B), which was primarily influenced by the type and position of the substituent attached to the phenyl group. In general, the shape of the absorption spectrum and the position of its maximum do not depend on the type of substituent, but on its place. The presence of chlorine at locant six on the benzene ring of 2-mercaptobenzothiazole (compound **2** and **5**) resulted in slight shifts toward higher wavelengths (bathochromic shift) compared to the compound with the same substituent at position five (**3** and **6**) (Figure 4C). The molar absorption coefficient (ε) in the **1**−**6** series varied between 8800 and 16,200 M^−1^·cm^−1^ (Table 4). The spectroscopic characteristics of the compounds studied are presented in Table 4.

### 3.4. Computational Details

**Figure 5:**
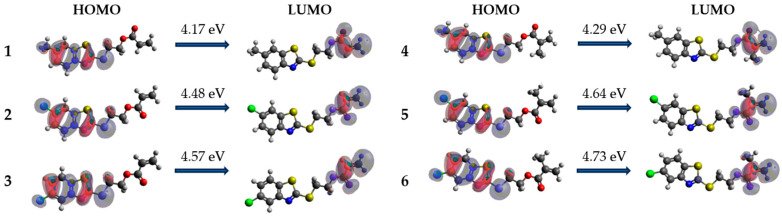
Frontier molecular diagram of 2-(2-benzothiazolylthio)ethyl acrylate (**1**–**3**) and methacrylate derivatives (**4**–**6**) in dichloromethane.

The HOMO (Highest Occupied Molecular Orbital) and LUMO (Lowest Unoccupied Molecular Orbital) values provide insights into the energy levels of molecular orbitals within a given molecule. This information is crucial for comprehending the chemical properties of molecules, reactivity, and behavior in chemical reactions. Similar to the previously tested compounds [42], the calculations indicated that the derivatives with electron-donating groups exhibited the highest reactivity (compounds **1** and **4**). Such molecules showed the lowest values of hardness and energy gaps (Table 5). Additionally, the higher energy gap values of 2-(2-benzothiazolylthio)ethyl methacrylate derivatives (**4**–**6**) suggest that they may be less reactive than their acrylic counterparts (**1**–**3**) (Figure 5). Notably, there was no evident impact of solvent polarity on the calculated parameters.

The activity of the photoinitiation of free-radical photopolymerization of the studied compounds will be confirmed by kinetic studies in a future paper.

## 4. Conclusions

The synthesis of novel 2-(2-benzothiazolylthio)ethyl acrylates (**1**–**3**) and methacrylates (**4**–**6**) is described here. The chemical structures of the synthesized compounds were confirmed by using various spectroscopic methods. Experimental spectroscopic data were effectively reproduced and complemented by quantum chemical DFT theoretical calculations. The study revealed that the replacement of the hydrogen atom in acrylate derivatives (R^3^ = H in Scheme 1) on the methyl group to methacrylate (R^3^ = CH_3_ in Scheme 1) had only a minor effect on their spectroscopic properties. In addition, it was shown that the solvent had a weak impact on the position of the absorption band, while the structure of the compound and the type and position of the substituent significantly influenced it. The substitution of the same substituent into the C6 (R^2^ in Scheme 1) position of the phenyl ring of the 2-mercaptobenzothiazole resulted in a slight shift toward a higher wavelength (bathochromic shift) when compared to the compound with substitution into the C5 (R^1^ in Scheme 1) position. The obtained HOMO and LUMO energy values indicated that 2-(2-benzothiazolylthio)ethyl acrylate derivatives (**1**–**3**) exhibited greater reactivity compared to 2-(2-benzothiazolylthio)ethyl methacrylate derivatives (**4**–**6**). Further investigations will be focused on studying the activity of new 2-mercaptobenzothiazole-based acrylates and methacrylates in photo-initiating systems for radical polymerization of acrylates.

## Figures and Tables

**Table 1:** ^1^H NMR chemical shifts for 2-(2-benzothiazolylthio)ethyl acrylate derivatives (**1**–**3**) and methacrylate (**4**–**6**) derivatives in 0.1–0.2 M solutions in CDCl_3_ (measured), CDCl_3_, and DMSO-d_6_ (italic, calculated) at 303 K.

Compound	Solvent	CH=CH_2_/C(CH_3_)=CH_2_	CH=CH_2_	C(CH_3_)=CH_2_	CH_2_–O	S–CH_2_
**1**	CHCl_3_	6.41; 5.82	6.11	-	4.53	3.63
	*CHCl_3_*	*7.24*; *6.48*	*6.58*	-	*4.42*	*3.47*
	*DMSO-d_6_*	*7.23*; *6.56*	*6.68*	-	*4.52*	*3.51*
**2**	CHCl_3_	6.34; 5.76	6.04	-	4.46	3.58
	*CHCl_3_*	*7.12*; *6.29*	*6.47*	-	*4.38*	*3.46*
	*DMSO-d_6_*	*7.02*; *6.37*	*6.59*	-	*4.44*	*3.53*
**3**	CHCl_3_	6.35; 5.75	6.04	-	4.47	3.60
	*CHCl_3_*	*7.12*; *6.41*	*6.44*	-	*4.40*	*3.48*
	*DMSO-d_6_*	*7.01*; *6.38*	*6.58*	-	*4.42*	*3.53*
**4**	CHCl_3_	6.03; 5.47	-	1.83	4.44	3.56
	*CHCl_3_*	*7.38*; *6.17*	-	*2.27*	*4.84*	*3.46*
	*DMSO-d_6_*	*7.36*; *6.24*	-	*2.30*	*4.93*	*3.49*
**5**	CHCl_3_	6.13; 5.58	-	1.94	4.55	3.69
	*CHCl_3_*	*6.81*; *6.12*	-	*2.22*	*4.34*	*3.61*
	*DMSO-d_6_*	*6.77*; *6.08*	-	*2.15*	*4.29*	*3.56*
**6**	CHCl_3_	6.04; 5.49	-	1.85	4.46	3.60
	*CHCl_3_*	*6.56*; *5.84*	-	*2.24*	*4.47*	*3.63*
	*DMSO-d_6_*	*6.70*; *5.93*	-	*2.14*	*4.42*	*3.57*

**Table 2:** ^13^C NMR chemical shifts of 2-(2-benzothiazolylthio)ethyl acrylate derivatives (**1**–**3**) and methacrylate derivatives (**4**–**6**) for 0.1–0.2 M solutions in CDCl_3_ (measured), CDCl_3_, and DMSO-d_6_ (italic, calculated) at 303 K.

Compound	Solvent	CH=CH_2_/C(CH_3_)=CH_2_	CH=CH_2_/C(CH_3_)=CH_2_	C=O	CH_2_–O	S–CH_2_
**1**	CHCl_3_	131.23	128.02	165.77	62.75	31.86
	*CHCl_3_*	*147.15*	*128.61*	*172.28*	*68.79*	*39.49*
	*DMSO-d_6_*	*147.79*	*128.92*	*173.92*	*69.25*	*39.60*
**2**	CHCl_3_	131.23	127.98	165.71	62.58	31.86
	*CHCl_3_*	*139.15*	*134.17*	*170.93*	*64.66*	*38.02*
	*DMSO-d_6_*	*142.74*	*135.31*	*175.04*	*66.37*	*39.31*
**3**	CHCl_3_	131.25	127.90	165.57	62.46	31.75
	*CHCl_3_*	*139.45*	*133.77*	*173.98*	*65.01*	*37.26*
	*DMSO-d_6_*	*142.79*	*135.30*	*175.07*	*66.44*	*39.34*
**4**	CHCl_3_	125.97	135.98	167.01	62.92	31.93
	*CHCl_3_*	*148.30*	*143.64*	*171.72*	*72.90*	*40.91*
	*DMSO-d_6_*	*148.57*	*144.15*	*173.44*	*73.27*	*40.98*
**5**	CHCl_3_	126.05	122.18	167.01	62.73	31.98
	*CHCl_3_*	*138.00*	*147.64*	*177.07*	*67.76*	*37.13*
	*DMSO-d_6_*	*137.10*	*146.94*	*175.34*	*66.24*	*35.53*
**6**	CHCl_3_	126.05	135.92	166.95	62.69	31.93
	*CHCl_3_*	*138.15*	*148.23*	*176.98*	*68.08*	*40.00*
	*DMSO-d_6_*	*136.40*	*146.98*	*175.24*	*66.38*	*39.53*

**Table 3:** Characteristic band strength values of the synthesized compounds.

Compounds	ν_s_ and ν_as_ (C–H)			ν_s_ and ν_as_ (C–H)	ν (C=O)	ν (C=C)
	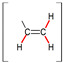	or	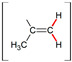	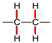		
**1**	3031–3425			2860–2951	1720	1632
**2**	3036–3435			2690–2946	1720	1633
**3**	3035–3435			2658–2947	1721	1634
**4**	2950–3021			2734–2922	1715	1636
**5**	2951–3021			2734–2922	1715	1636
**6**	3066–3085			2836–2978	1714	1636

**Table 4:** Measured and calculated spectroscopic data for 2-(2-benzothiazolylthio)ethyl acrylate derivatives (**1**–**3**) and methacrylate derivatives (**4**–**6**) in selected solvents of different polarities.

Compound	Solvent	λ_ab_ (nm) (Measured)	λ_ab_ (nm) (Calculated)	ε (×10^4^, M^−1^ · cm^−1^)
**1**	DCM	283	278	1.21
	MeOH	282	278	1.14
	DMSO	285	279	1.21
**2**	DCM	285	282	1.21
	MeOH	283	281	1.32
	DMSO	285	282	1.15
**3**	DCM	279	283	1.16
	MeOH	278	282	0.88
	DMSO	280	283	1.45
**4**	DCM	285	279	1.42
	MeOH	282	278	1.30
	DMSO	285	279	1.17
**5**	DCM	285	282	1.62
	MeOH	283	281	1.48
	DMSO	286	282	1.56
**6**	DCM	279	281	1.32
	MeOH	277	281	1.10
	DMSO	279	282	1.23

**Table 5:** The values of hardness (η), energy gap, and energies of HOMO and LUMO orbitals estimated for all compounds.

Compound	Solvent	HOMO (eV)	LUMO (eV)	Energy Gap (eV)	η (eV)
**1**	DCM	−6.1358	−1.9627	4.1731	2.0865
	MeOH	−6.1415	−1.9508	4.1908	2.0954
	DMSO	−6.1423	−1.9497	4.1927	2.0963
**2**	DCM	−6.2863	−1.8019	4.4844	2.2422
	MeOH	−6.2822	−1.7916	4.4906	2.2453
	DMSO	−6.2819	−1.7905	4.4914	2.2457
**3**	DCM	−6.3704	−1.8044	4.5660	2.2830
	MeOH	−6.3628	−1.7927	4.5701	2.2850
	DMSO	−6.3622	−1.7913	4.5709	2.2855
**4**	DCM	−6.1312	−1.8419	4.2893	2.1446
	MeOH	−6.1383	−1.8422	4.2961	2.1480
	DMSO	−6.1394	−1.8422	4.2972	2.1486
**5**	DCM	−6.2836	−1.6389	4.6446	2.3223
	MeOH	−6.2808	−1.6357	4.6452	2.3226
	DMSO	−6.2806	−1.6354	4.6452	2.3226
**6**	DCM	−6.3696	−1.6416	4.7279	2.3640
	MeOH	−6.3606	−1.6365	4.7241	2.3621
	DMSO	−6.3598	−1.6362	4.7236	2.3618

## Data Availability

The RepOD Open Data Repository (https://doi.org/10.18150/R9VGW9) contains raw data and metadata related to the spectral characteristics of 2-mercaptobenzothiazole derivatives.

## Supplementary Materials

The following supporting information can be downloaded at: https://www.mdpi.com/article/10.3390/ma17010246/s1. Figure S1. ^1^H NMR spectrum (400 MHz) of 2-(2-(6-methylbenzothiazolyl)thio)ethyl acrylate (**1**) in CDCl_3_; Figure S2. ^13^C NMR spectrum (400 MHz) of 2-(2-(6-methylbenzothiazolyl)thio)ethyl acrylate (**1**) in CDCl_3_; Figure S3. ^1^H NMR spectrum (400 MHz) of 2-(2-(6-chlorobenzothiazolyl)thio)ethyl acrylate (**2**) in CDCl_3_; Figure S4. ^13^C NMR spectrum (400 MHz) of 2-(2-(6-chlorobenzothiazolyl)thio)ethyl acrylate (**2**) in CDCl_3_; Figure S5. ^1^H NMR spectrum (400 MHz) of 2-(2-(5-chlorobenzothiazolyl)thio)ethyl acrylate (**3**) in CDCl_3_; Figure S6. ^13^C NMR spectrum (400 MHz) of 2-(2-(5-chlorobenzothiazolyl)thio)ethyl acrylate (**3**) in CDCl_3_; Figure S7. ^1^H NMR spectrum (400 MHz) of 2-(2-(6-methylbenzothiazolyl)thio)ethyl methacrylate (**4**) in CDCl_3_; Figure S8. ^13^C NMR spectrum (400 MHz) of 2-(2-(6-methylbenzothiazolyl)thio)ethyl methacrylate (**4**) in CDCl_3_; Figure S9. ^1^H NMR spectrum (400 MHz) of 2-(2-(6-chlorobenzothiazolyl)thio)ethyl methacrylate (**5**) in CDCl_3_; Figure S10. ^13^C NMR spectrum (400 MHz) of 2-(2-(6-chlorobenzothiazolyl)thio)ethyl methacrylate (**5**) in CDCl_3_; Figure S11. ^1^H NMR spectrum (400 MHz) of 2-(2-(5-chlorobenzothiazolyl)thio)ethyl methacrylate (**6**) in CDCl_3_; Figure S12. ^13^C NMR spectrum (400 MHz) of 2-(2-(5-chlorobenzothiazolyl)thio)ethyl methacrylate (**6**) in CDCl_3_; Figure S13. Chromatogram of 2-(2-(6-methylbenzothiazolyl)thio)ethyl acrylate (**1**); Figure S14. Mass spectrum of 2-(2-(6-methylbenzothiazolyl)thio)ethyl acrylate (**1**); Figure S15. Chromatogram of 2-(2-(6-chlorobenzothiazolyl)thio)ethyl acrylate (**2**); Figure S16. Mass spectrum of 2-(2-(6-chlorobenzothiazolyl)thio)ethyl acrylate (**2**); Figure S17. Chromatogram of 2-(2-(5-chlorobenzothiazolyl)thio)ethyl acrylate (**3**); Figure S18. Mass spectrum of 2-(2-(5-chlorobenzothiazolyl)thio)ethyl acrylate (**3**); Figure S19. Chromatogram of 2-(2-(6-methylbenzothiazolyl)thio)ethyl methacrylate (**4**); Figure S20. Mass spectrum of 2-(2-(6-methylbenzothiazolyl)thio)ethyl methacrylate (**4**); Figure S21. Chromatogram of 2-(2-(6-chlorobenzothiazolyl)thio)ethyl methacrylate (**5**); Figure S22. Mass spectrum of 2-(2-(6-chlorobenzothiazolyl)thio)ethyl methacrylate (**5**); Figure S23. Chromatogram of 2-(2-(5-chlorobenzothiazolyl)thio)ethyl meyhacrylate (**6**); Figure S24. Mass spectrum of 2-(2-(5-chlorobenzothiazolyl)thio)ethyl methacrylate (**6**).
