# Supplementary material for: Design, Synthesis, and Spectral Properties of Novel 2-Mercaptobenzothiazole Derivatives
Source: Materials (Basel). 2024 Jan 2;17(1):246. doi: 10.3390/ma17010246 (PMC10779768; doi:10.3390/ma17010246)
Supplement: Supplementary file 1 [file materials-17-00246-s001.zip › materials-2772712-supplementary.pdf]

Design, Synthesis, and Spectral Properties of Novel 2-Mercaptobenzothiazole Derivatives

Agnieszka Skotnicka *, Janina Kabatc-Borcz

Faculty of Chemical Technology and Engineering, Bydgoszcz University of Science and Technology, Seminaryjna 3, 85-326 Bydgoszcz, Poland

*Correspondence: askot@pbs.edu.pl

Supporting Information

Table of contents

S1	^{1}H NMR spectrum (400 MHz) of 2-(2-(6-methylbenzothiazolyl)thio)ethyl acrylate (**1**) in $CDCl_3$	s 3
S2	^{13}C NMR spectrum (400 MHz) of 2-(2-(6-methylbenzothiazolyl)thio)ethyl acrylate (**1**) in $CDCl_3$ ……………………………………………………………………………………………	s 3
S3	^{1}H NMR spectrum (400 MHz) of 2-(2-(6-chlorobenzothiazolyl)thio)ethyl acrylate (**2**) in $CDCl_3$	s 4
S4	^{13}C NMR spectrum (400 MHz) of 2-(2-(6-chlorobenzothiazolyl)thio)ethyl acrylate (**2**) in $CDCl_3$	s 4
S5	^{1}H NMR spectrum (400 MHz) of 2-(2-(5-chlorobenzothiazolyl)thio)ethyl acrylate (**3**) in $CDCl_3$	s 5
S6	^{13}C NMR spectrum (400 MHz) of 2-(2-(5-chlorobenzothiazolyl)thio)ethyl acrylate (**3**) in $CDCl_3$	s 5
S7	^{1}H NMR spectrum (400 MHz) of 2-(2-(6-methylbenzothiazolyl)thio)ethyl methacrylate (**4**) in $CDCl_3$ ……………………………………………………………………………………………	s 6
S8	^{13}C NMR spectrum (400 MHz) of 2-(2-(6-methylbenzothiazolyl)thio)ethyl methacrylate (**4**) in $CDCl_3$ ……………………………………………………………………………………………	s 6
S9	^{1}H NMR spectrum (400 MHz) of 2-(2-(6-chlorobenzothiazolyl)thio)ethyl methacrylate (**5**) in $CDCl_3$ ……………………………………………………………………………………………	s 7
S10	^{13}C NMR spectrum (400 MHz) of 2-(2-(6-chlorobenzothiazolyl)thio)ethyl methacrylate (**5**) in $CDCl_3$ ……………………………………………………………………………………………	s 7
S11	^{1}H NMR spectrum (400 MHz) of 2-(2-(5-chlorobenzothiazolyl)thio)ethyl methacrylate (**6**) in $CDCl_3$ ……………………………………………………………………………………………	s 8
S12	^{13}C NMR spectrum (400 MHz) of 2-(2-(5-chlorobenzothiazolyl)thio)ethyl methacrylate (**6**) in $CDCl_3$ ……………………………………………………………………………………………	s 8
S13	Chromatogram of 2-(2-(6-methylbenzothiazolyl)thio)ethyl acrylate (**1**) ………………………..	s 9
S14	Mass spectrum of 2-(2-(6-methylbenzothiazolyl)thio)ethyl acrylate (**1**) ………………………...	s 9
S15	Chromatogram of 2-(2-(6-chlorobenzothiazolyl)thio)ethyl acrylate (**2**) ………………………...	s 9
S16	Mass spectrum of 2-(2-(6-chlorobenzothiazolyl)thio)ethyl acrylate (**2**) …………………………	s 9
S17	Chromatogram of 2-(2-(5-chlorobenzothiazolyl)thio)ethyl acrylate (**3**) ………………………...	s 10
S18	Mass spectrum of 2-(2-(5-chlorobenzothiazolyl)thio)ethyl acrylate (**3**) …………………………	s 10
S19	Chromatogram of 2-(2-(6-methylbenzothiazolyl)thio)ethyl methacrylate (**4**) …………..……..	s 10

S20 Mass spectrum of 2-(2-(6-methylbenzothiazolyl)thio)ethyl methacrylate (**4**) …………………... s 10
S21 Chromatogram of 2-(2-(6-chlorobenzothiazolyl)thio)ethyl acrylate (**5**) ………………………... s 11
S22 Mass spectrum of 2-(2-(6-chlorobenzothiazolyl)thio)ethyl acrylate (**5**) ………………………… s 11
S23 Chromatogram of 2-(2-(5-chlorobenzothiazolyl)thio)ethyl acrylate (**6**) ………………………... s 11
S24 Mass spectrum of 2-(2-(5-chlorobenzothiazolyl)thio)ethyl acrylate (**6**) ………………………… s 11

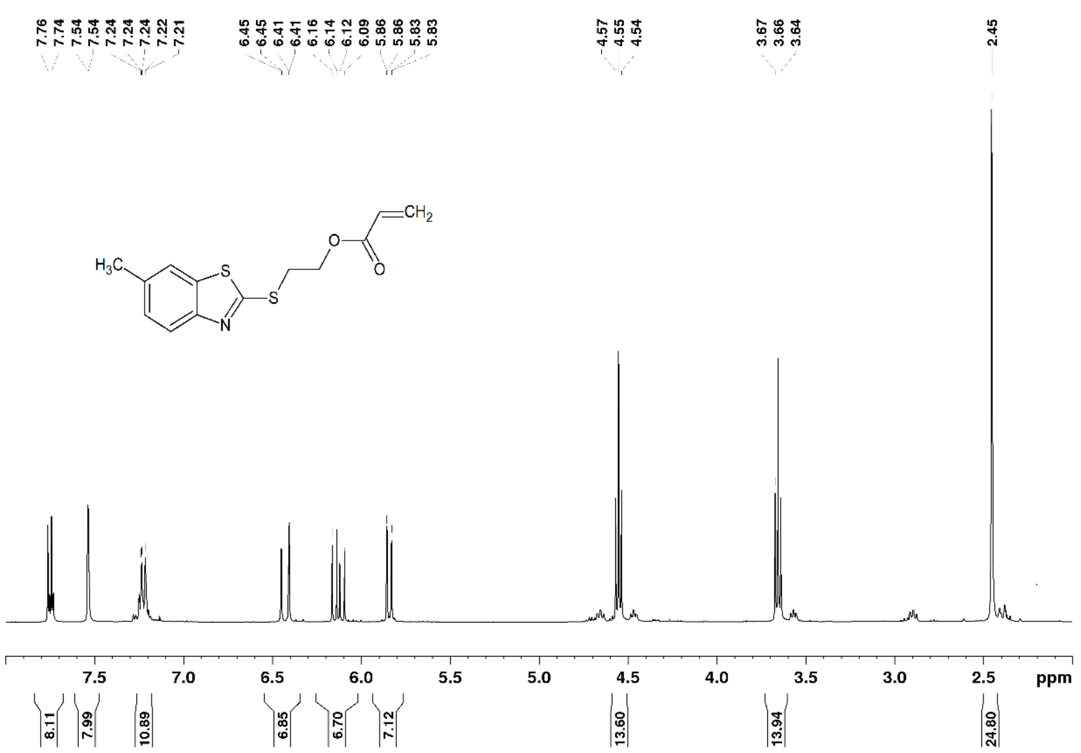

Figure S1. ^{1}H NMR spectrum (400 MHz) of 2-(2-(6-methylbenzothiazolyl)thio)ethyl acrylate (**1**) in $CDCl_3$.

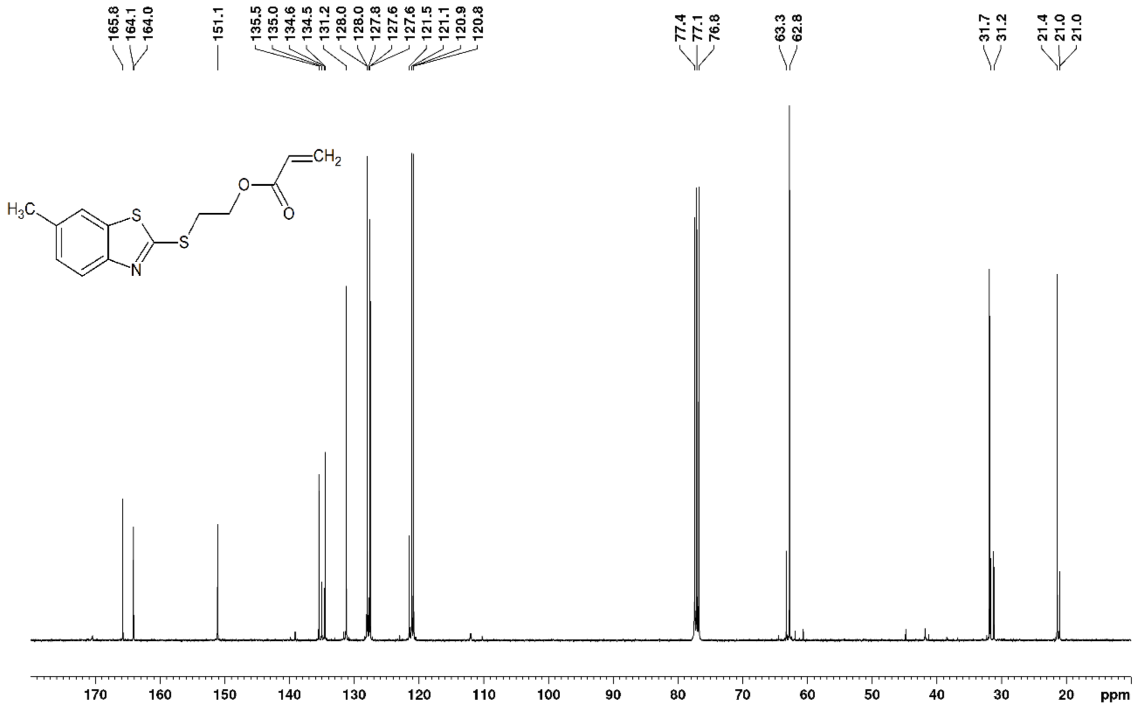

Figure S2. ^{13}C NMR spectrum (400 MHz) of 2-(2-(6-methylbenzothiazolyl)thio)ethyl acrylate (**1**) in $CDCl_3$.

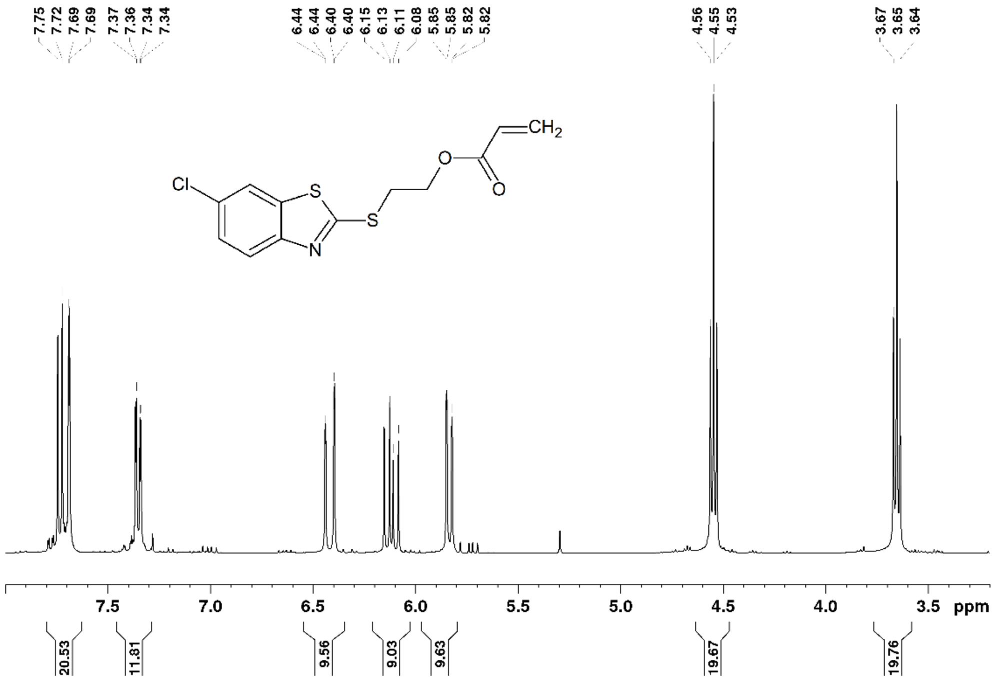

Figure S3. ^{1}H NMR spectrum (400 MHz) of 2-(2-(6-chlorobenzothiazolyl)thio)ethyl acrylate **(2)** in $CDCl_3$.

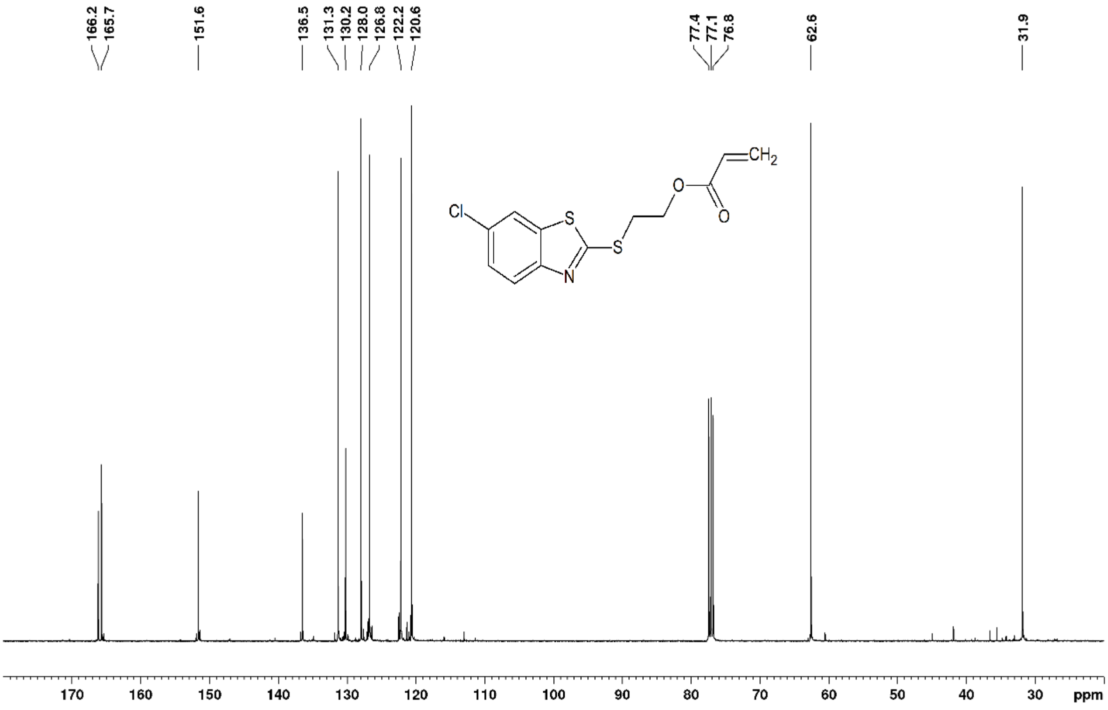

Figure S4. ^{13}C NMR spectrum (400 MHz) of 2-(2-(6-chlorobenzothiazolyl)thio)ethyl acrylate (**2**) in $CDCl_3$.

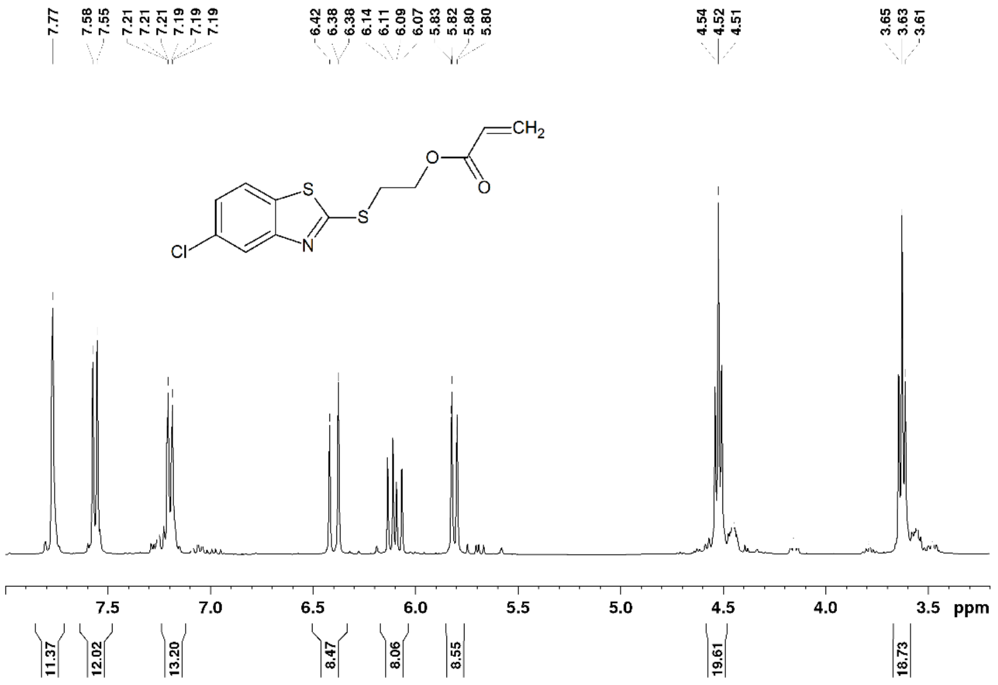

Figure S5. ^{1}H NMR spectrum (400 MHz) of 2-(2-(5-chlorobenzothiazolyl)thio)ethyl acrylate (**3**) in $CDCl_3$.

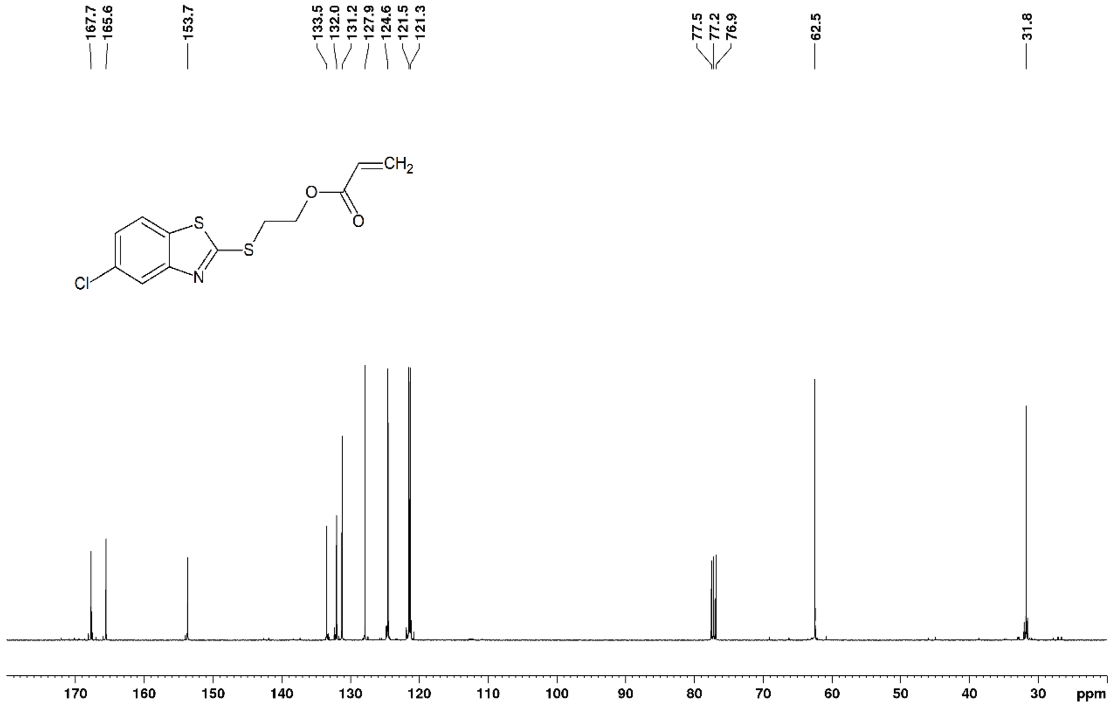

Figure S6. ^{13}C NMR spectrum (400 MHz) of 2-(2-(5-chlorobenzothiazolyl)thio)ethyl acrylate (**3**) in $CDCl_3$.

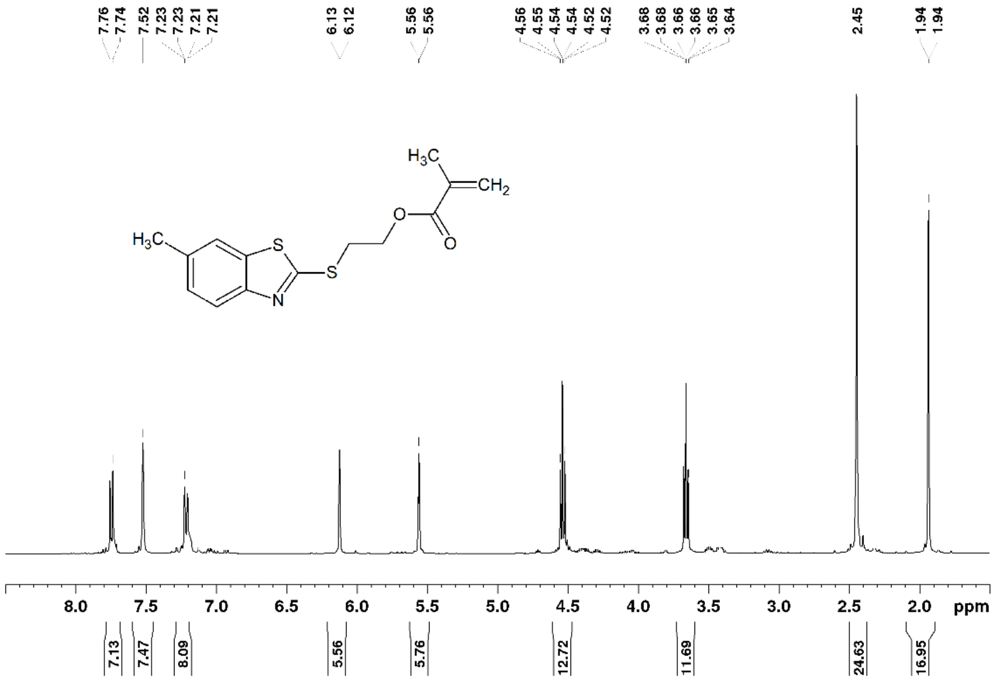

Figure S7. ^{1}H NMR spectrum (400 MHz) of 2-(2-(6-methylbenzothiazolyl)thio)ethyl methacrylate (**4**) in $CDCl_3$.

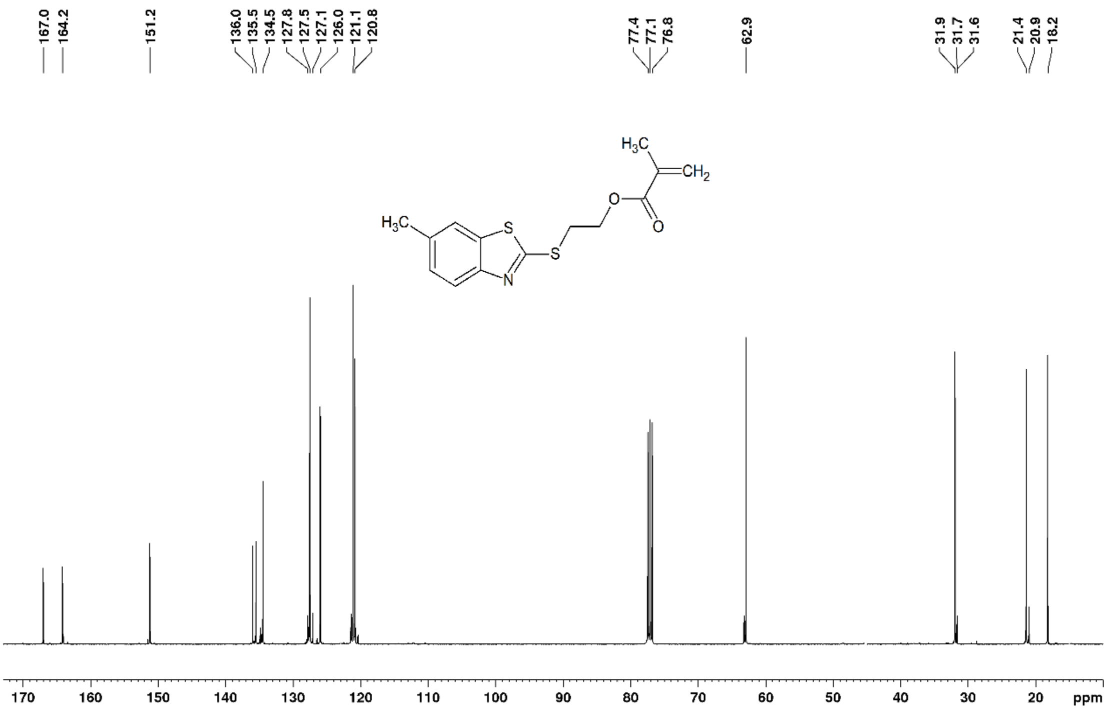

Figure S8.^{13}C NMR spectrum (400 MHz) of 2-(2-(6-methylbenzothiazolyl)thio)ethyl methacrylate (**4**) in $CDCl_3$.

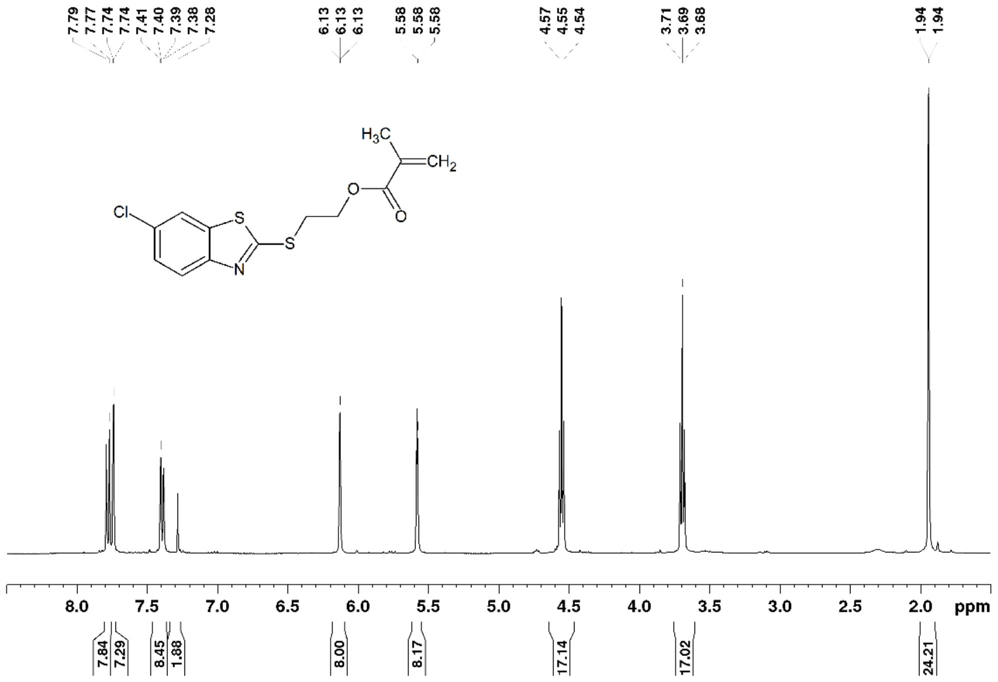

Figure S9. ^{1}H NMR spectrum (400 MHz) of 2-(2-(6-chlorobenzothiazolyl)thio)ethyl methacrylate (**5**) in $CDCl_3$.

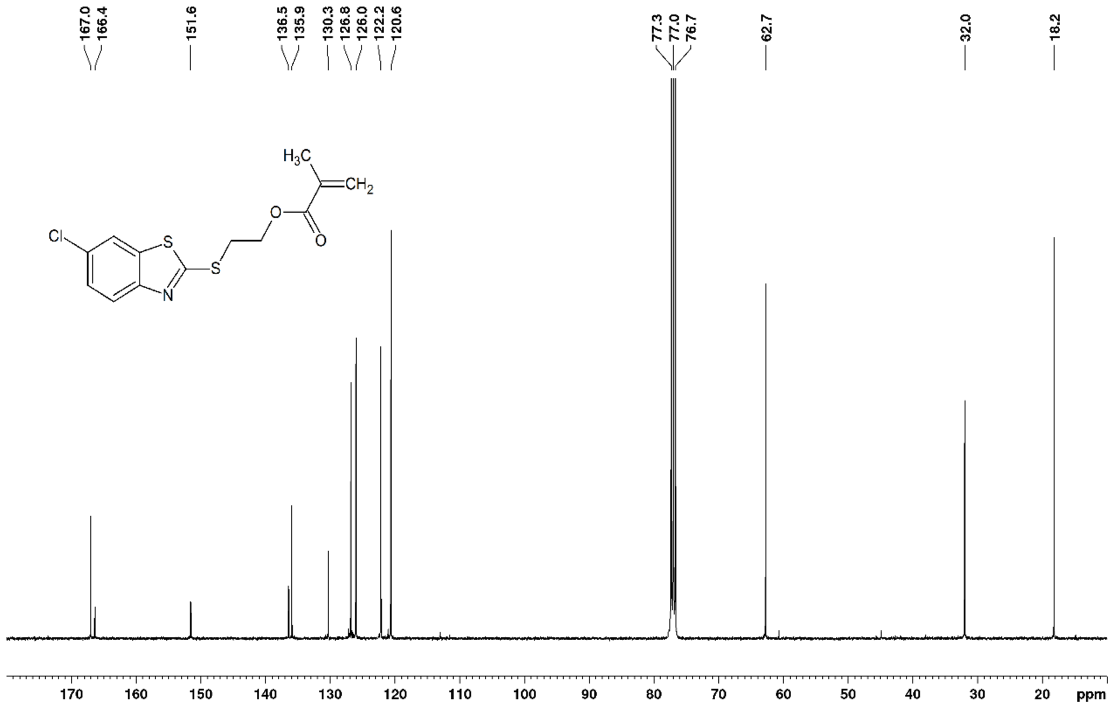

Figure S10.^{13}C NMR spectrum (400 MHz) of 2-(2-(6-chlorobenzothiazolyl)thio)ethyl methacrylate (**5**) in $CDCl_3$

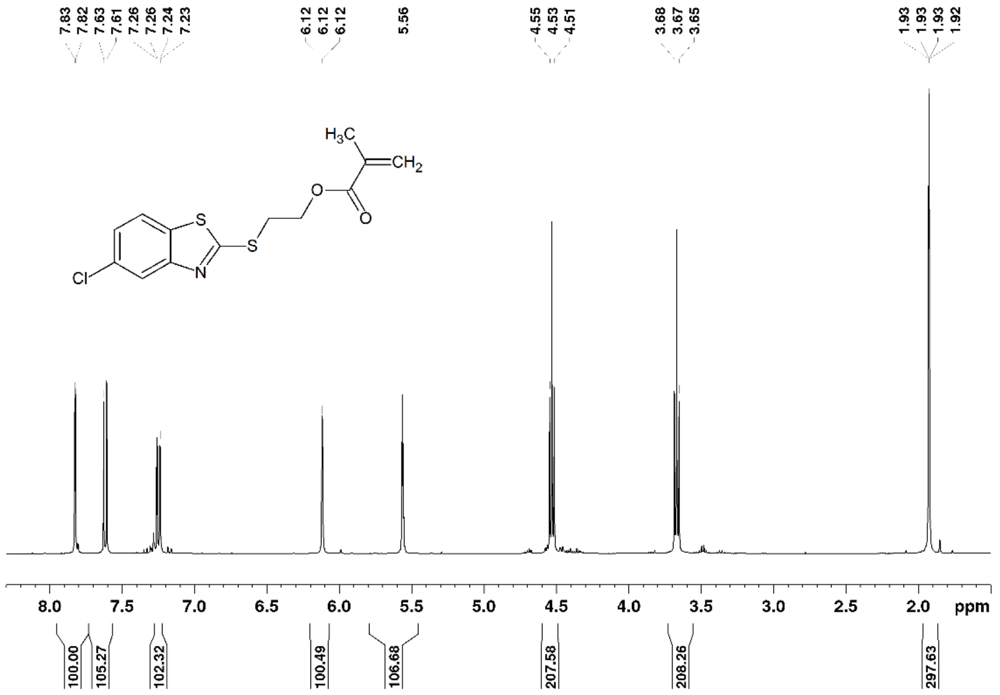

Figure S11.^{1}H NMR spectrum (400 MHz) of 2-(2-(5-chlorobenzothiazolyl)thio)ethyl methacrylate (**6**) in $CDCl_3$.

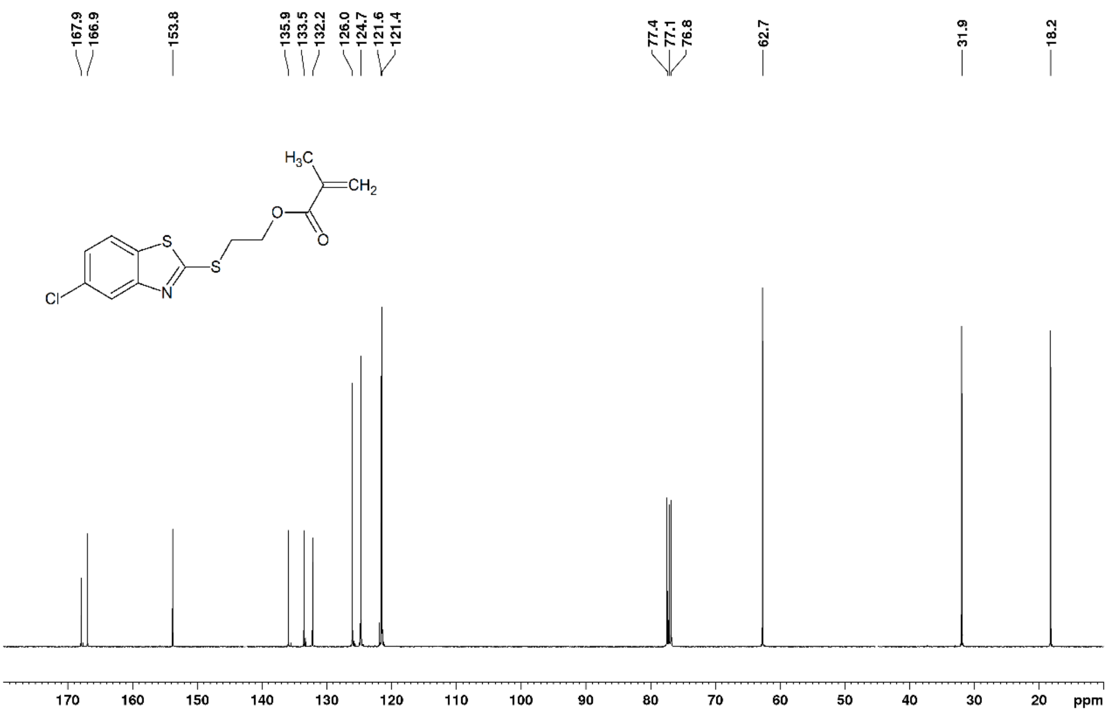

Figure S12.^{13}C NMR spectrum (400 MHz) of 2-(2-(5-chlorobenzothiazolyl)thio)ethyl methacrylate (**6**) in $CDCl_3$

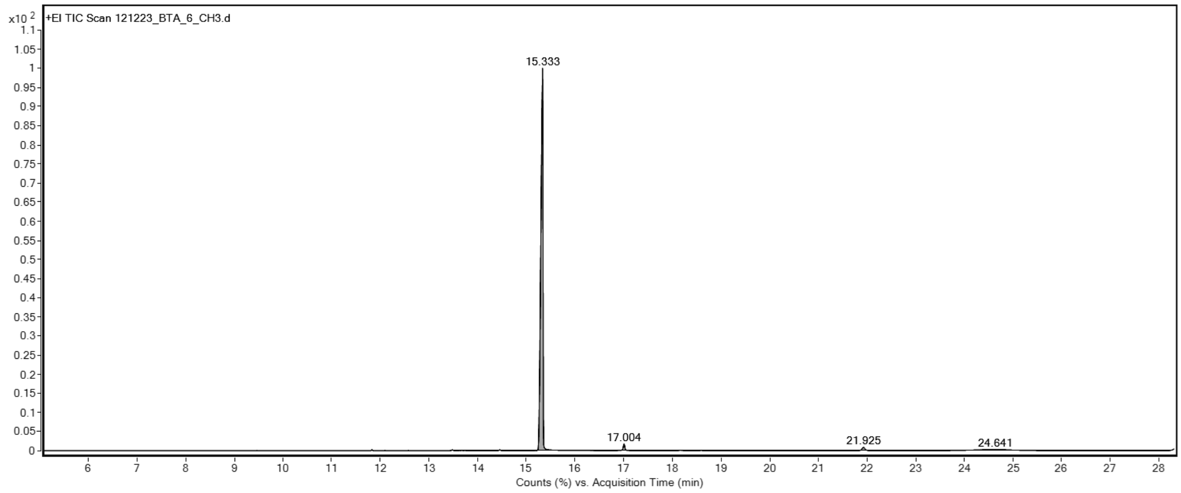

Figure S13. Chromatogram of 2-(2-(6-methylbenzothiazolyl)thio)ethyl acrylate (**1**).

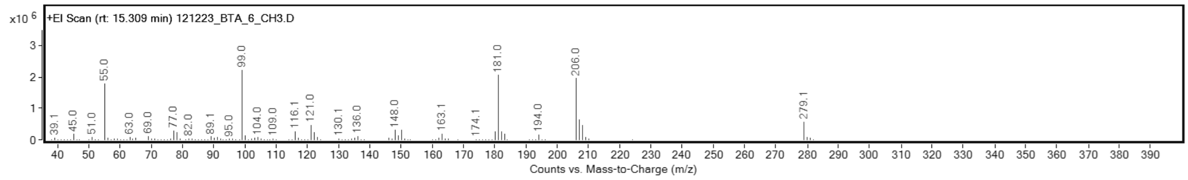

Figure S14. Mass spectrum of 2-(2-(6-methylbenzothiazolyl)thio)ethyl acrylate (**1**).

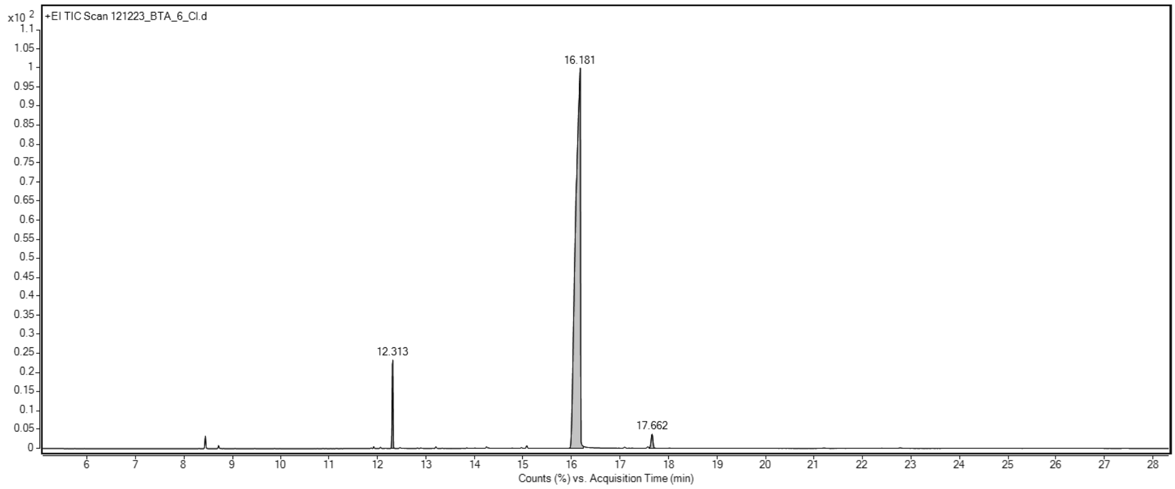

Figure S15. Chromatogram of 2-(2-(6-chlorobenzothiazolyl)thio)ethyl acrylate (**2**).

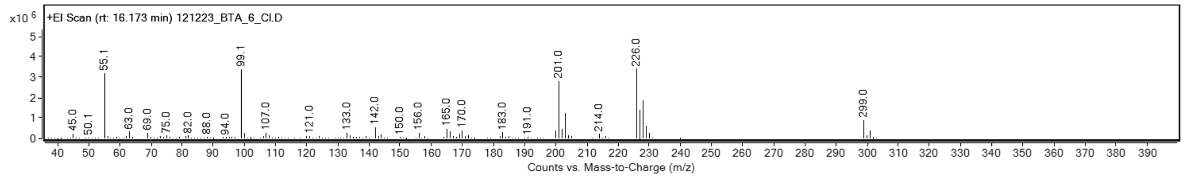

Figure S16. Mass spectrum of 2-(2-(6-chlorobenzothiazolyl)thio)ethyl acrylate (**2**).

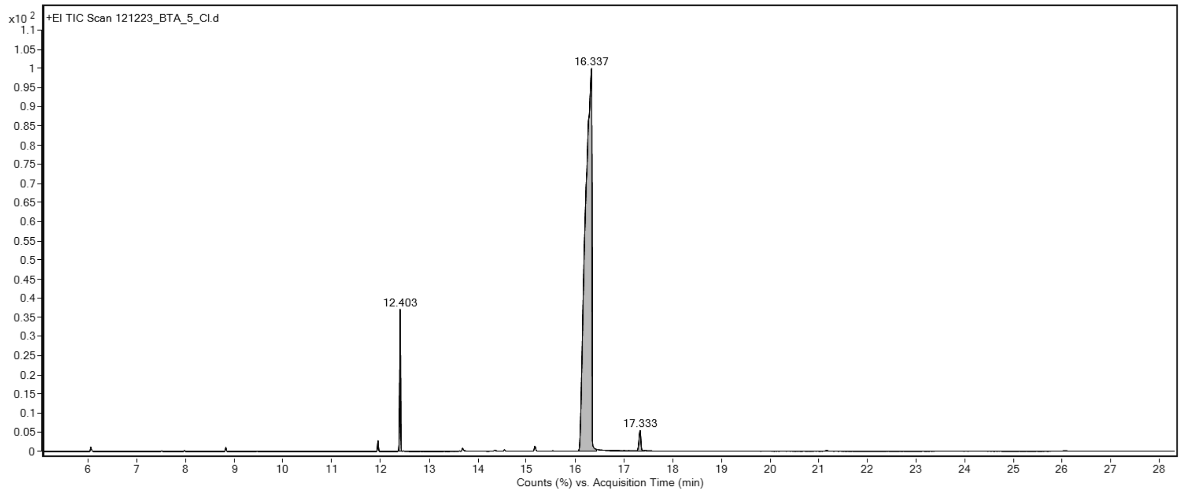

Figure S17. Chromatogram of 2-(2-(5-chlorobenzothiazolyl)thio)ethyl acrylate (**3**).

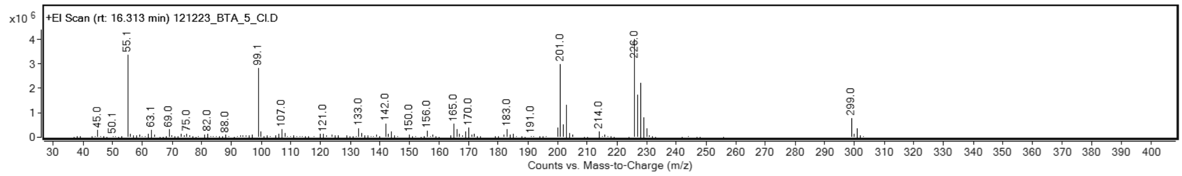

Figure S18. Mass spectrum of 2-(2-(5-chlorobenzothiazolyl)thio)ethyl acrylate (**3**).

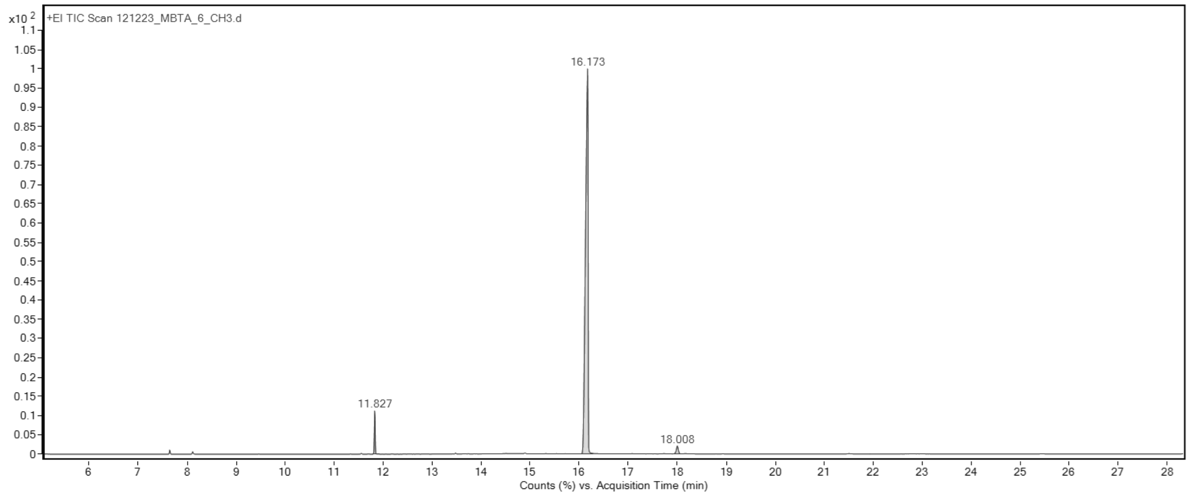

Figure S19. Chromatogram of 2-(2-(6-methylbenzothiazolyl)thio)ethyl methacrylate (**4**).

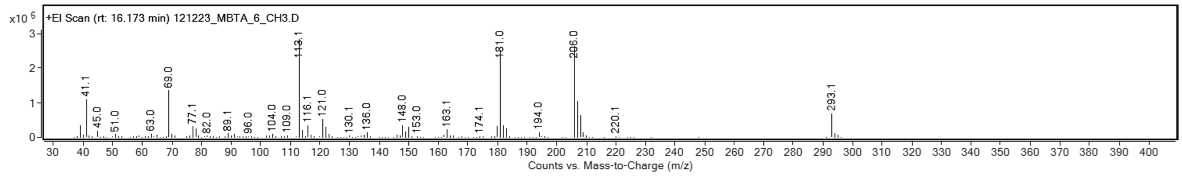

Figure S20. Mass spectrum of 2-(2-(6-methylbenzothiazolyl)thio)ethyl methacrylate (**4**).

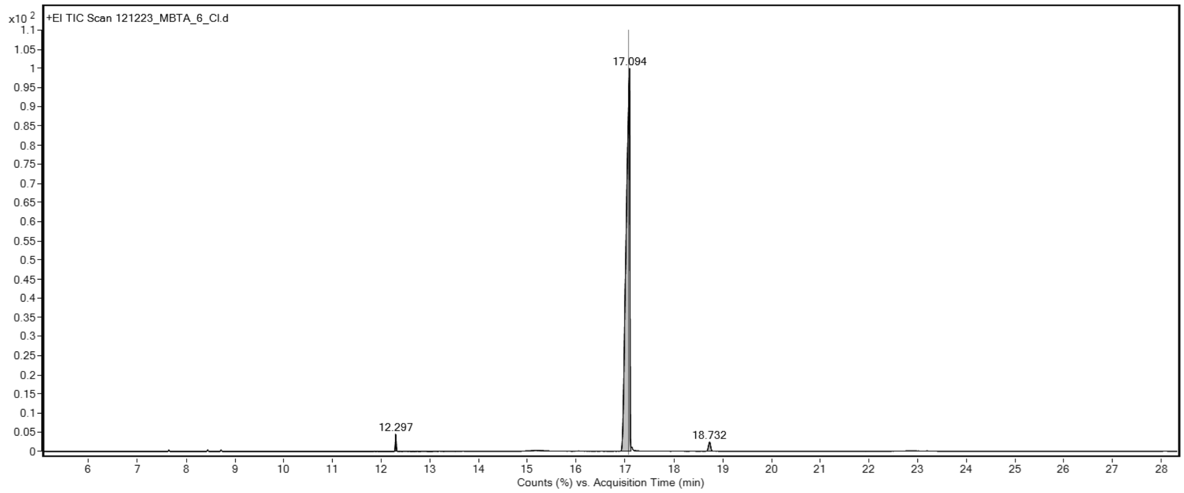

Figure S21. Chromatogram of 2-(2-(6-chlorobenzothiazolyl)thio)ethyl methacrylate (**5**).

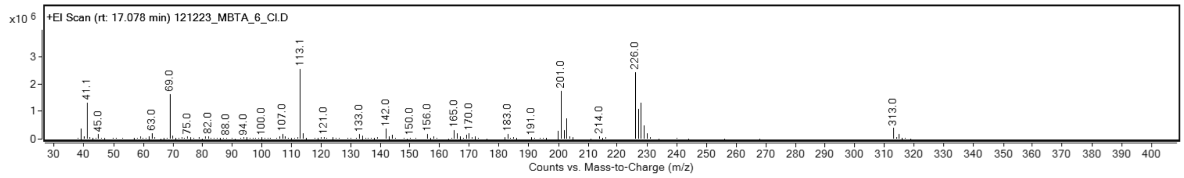

Figure S22. Mass spectrum of 2-(2-(6-chlorobenzothiazolyl)thio)ethyl methacrylate (**5**).

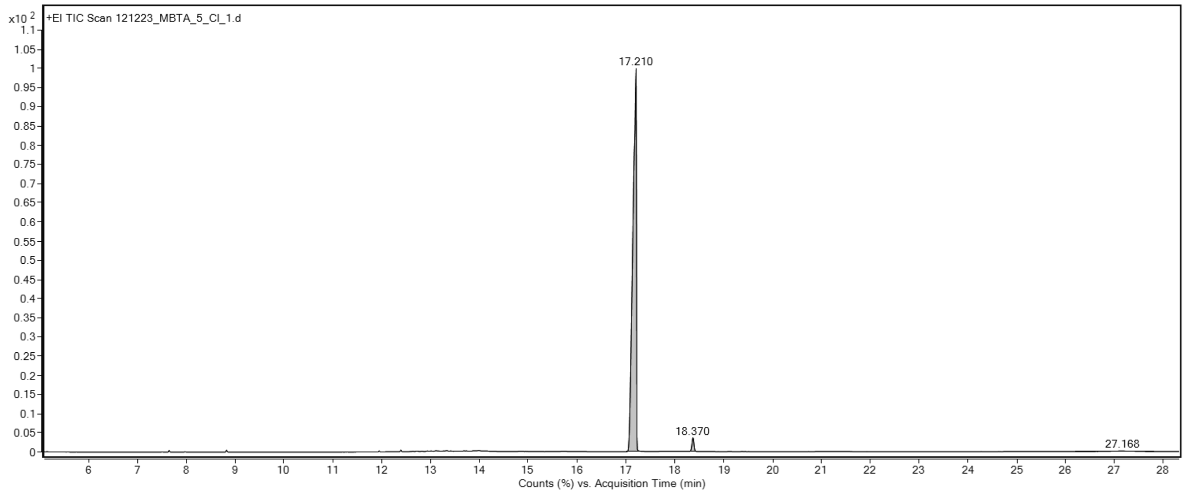

Figure S23. Chromatogram of 2-(2-(5-chlorobenzothiazolyl)thio)ethyl methacrylate (**6**).

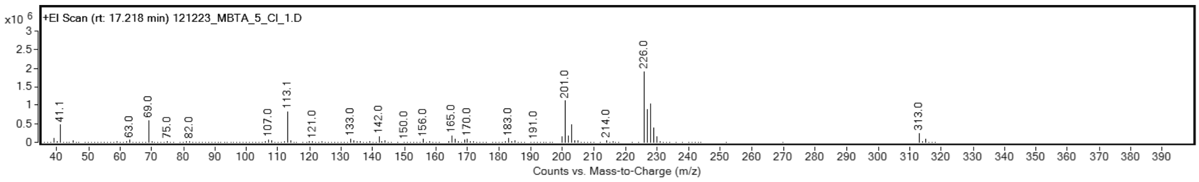

Figure S24. Mass spectrum of 2-(2-(5-chlorobenzothiazolyl)thio)ethyl methacrylate (**6**).
